# Using implementation science to promote evidence-based nutritional care in healthcare settings: A mixed-methods systematic review

**DOI:** 10.1016/j.ijnsa.2025.100414

**Published:** 2025-08-24

**Authors:** Jerome Molle, Joris Agnel, Sebastien Colson, Audrey Chays-Amania

**Affiliations:** a Faculty of Medical and Paramedical Sciences, Nursing School, CEReSS, Aix-Marseille University, Marseille, France; b Intercommunal Hospital Center of the Southern Alps, Gap, France; c Faculty of Medical and Paramedical Sciences, Nursing School, CEReSS, APHM, Aix-Marseille University, Marseille, France

**Keywords:** Nutrition, Evidence-based, Implementation strategies, Tailored strategies, Multifaceted interventions, Implementation outcomes, Clinical outcomes

## Abstract

**Background:**

Up to half of hospital inpatients are malnourished, a condition that prolongs recovery, increases complications and raises healthcare costs. Although evidence-based nutritional care can mitigate these effects, its routine implementation remains inconsistent.

**Objective:**

To synthesise and evaluate current evidence on the implementation and the clinical effectiveness of strategies designed to promote evidence-based nutritional care practices within healthcare settings.

**Methods:**

A mixed-methods systematic review followed the Joanna Briggs Institute guidelines. Fifteen databases were searched for studies published between January 2015 and January 2025 that evaluated implementation strategies targeting evidence-based nutrition care for any patient or healthcare professional group. Two reviewers independently screened records extracted data and applied the Mixed-Methods Appraisal Tool. Because study designs, contexts and outcome metrics varied, findings were integrated narratively using the Consolidated Framework for Implementation Research, the Expert Recommendations for Implementing Change taxonomy and the Implementation Outcomes Framework.

**Results:**

Twenty-nine primary studies involving 1624 healthcare professionals and 13,523 patients were included. All interventions were multifaceted and tailored to the context. The most frequent components were staff education (97 %), audit with feedback (93 %), stakeholder engagement structures (62 %), and adaptations to electronic or physical workflows (28 %). Principal barriers experienced by healthcare professionals comprised scarce resources, fragmented communication, inadequate infrastructure, and knowledge deficits; key facilitators were visible leadership, standardised communication tools, iterative planning, and a strong evidence base. Fidelity of intervention delivery by healthcare professionals was consistently high (median ≥ 80 %); acceptability exceeded 70 % in all studies that assessed it, and feasibility was rated favourably. Where measured, penetration and sustainability were moderate but positive. Service outcomes improved across settings, including earlier initiation of nutrition therapy, greater dietary adequacy, and fewer treatment interruptions or nutrition-related complications. Patient-level benefits comprised reduced weight loss, improved nutritional status, better health-related quality of life, and higher satisfaction.

**Conclusion:**

Context-sensitive, multifaceted implementation strategies, particularly those coupling education, audit-feedback, leadership engagement, and system redesign, can improve the uptake and impact of evidence-based nutritional care. Future studies should employ standardised frameworks, extended follow-up, and rigorous evaluation designs to assess sustainability and inform large-scale implementation.

**PROSPERO registration:**

Not registered.

**What is already known**

• Up to half of hospitalised patients are malnourished, leading to higher complication rates and costs
• Evidence-based nutritional care improves intake and recovery but are unevenly uptake
• Prior reviews catalogue barriers and facilitators without linking strategies to measurable outcomes

**What this paper adds**

• Demonstrates that theory-driven, locally tailored, multifaceted strategies achieve fidelity and acceptability among healthcare professionals in evidence-based nutritional care implementation
• Shows consistent gains in timeliness and adequacy of nutrition therapy, with fewer interruptions to patients’ nutrition therapy and fewer nutrition-related complications
• Provides an integrated map of implementation determinants, strategies and outcomes to guide context-responsive scale-up of evidence-based nutrition care practices

Alt-text: Unlabelled box

## 1 Background

Malnutrition is a prevalent issue among hospitalised patients, with studies estimating its occurrence at admission to range between 30 % and 50 % (Pradelli et al., 2023; Saijo et al., 2024; Van Vliet et al., 2020). Moreover, among those well-nourished at admission, approximately 30 % become malnourished before discharge, while 80 % of those who were initially malnourished remained in that condition throughout their stay (Botero et al., 2024; Van Vliet et al., 2020). Malnutrition is defined as a subacute or chronic nutritional state characterised by varying degrees of undernutrition or overnutrition combined with inflammatory activity, leading to alterations in body composition and diminished functional capacity (Keller et al., 2020). Significantly, hospital malnutrition is associated with a range of adverse outcomes, such as delayed wound healing, increased risk of complications and infections, prolonged length of stay, higher readmission rates, and elevated mortality (Botero et al., 2024; Compher et al., 2024). These consequences negatively affect patients' quality of life and contribute to higher healthcare costs (Schuetz et al., 2021; Sulo et al., 2021).

Given these serious consequences, an evidence-based approach to nutritional care is imperative (Kaegi-Braun et al., 2020). This approach integrates the best available evidence with clinical expertise, patient and family preferences, and contextual resources to guide decision-making in clinical practice (Chays‐Amania et al., 2024). In nutrition, the overarching objective is to prevent, resolve, or manage patients’ nutritional challenges effectively (Johnston et al., 2019). Converging evidence demonstrates the value of a systematic process, starting with the screening of all inpatients for nutritional risk upon hospital admission, followed by a comprehensive assessment and the initiation of individualised nutritional support for at-risk patients (Kaegi-Braun et al., 2021; Schuetz et al., 2019). The nutrition care process (NCP) serves as a structured, step-by-step framework for delivering nutritional care, encompassing malnutrition risk screening, assessment, diagnosis, care planning, nutrition therapy, monitoring, evaluation, and documentation (Cederholm et al., 2017). Despite the mounting evidence (Keller et al., 2021b; Neale and Tapsell, 2019) and the abundance of guidelines (Muscaritoli et al., 2021; Singer et al., 2023; Volkert et al., 2022; Weimann et al., 2021; Wunderle et al., 2024), significant gaps persist in routine practice (Findlay et al., 2020a; Wong et al., 2023).

Implementation science offers a path forward, rigorously evaluating methods to integrate evidence into practice (Eccles and Mittman, 2006). By examining strategies, contextual factors, mechanisms, and outcomes with robust methodologies, it generates actionable and generalisable insights to improve healthcare systems (Wilson et al., 2024). In nutrition, this discipline is increasingly recognised for its potential to close the research-practice gap and promote sustainable improvements in patient outcomes (Brown et al., 2021; Keller et al., 2021b).

Over the past two decades, the proliferation of theories, models, and frameworks has provided researchers and practitioners with an integrated set of conceptually distinct yet mutually reinforcing tools to address each core implementation process (Wang et al., 2023). Nilsen's (2015) five‐category taxonomy, comprising process models, determinant frameworks, classic theories, implementation theories, and evaluation frameworks, serves as a practical guide for matching research questions to the most appropriate conceptual instrument. To build cumulative knowledge, it is imperative that studies employ transparent, theory‐driven methods (Lengnick-Hall et al., 2023; Moullin et al., 2020). In line with this principle, our review is structured around three established frameworks that correspond directly to these functions: the Consolidated Framework for Implementation Research (CFIR) for determinants (Damschroder et al., 2022), the Expert Recommendations for Implementing Change (ERIC) compilation of strategies (Powell et al., 2015; Waltz et al., 2015), and the Implementation Outcomes Framework (IOF) for evaluation (Proctor et al., 2011). Together, they form a unified backbone for our inquiry (Table 1).

**Table 1:** Comparative summary of selected implementation frameworks.

Framework	Purpose	Core components & definitions
**CFIR** (Determinant framework)	Identify multilevel barriers and facilitators	5 domains of 39 constructs to assess contextual determinants: • Innovation – key attributes of what is being implemented (evidence strength, adaptability) • Outer setting – external influences (policy, peer pressure) • Inner setting – organisational context (culture, resources) • Individuals – characteristics of people involved (knowledge, self‐efficacy) • Implementation process – steps and strategies (planning, engaging, evaluating)
**ERIC** (Strategy taxonomy)	Catalogue and tailor actionable strategies	9 clusters of 73 discrete strategies grouped to facilitate targeted action: • Evaluative & iterative approaches • Interactive assistance • Adaptation to context • Stakeholder relationships • Training & education • Clinician support • Consumer engagement • Financial incentives • Infrastructure change
**IOF** (Evaluation framework)	Define and measure implementation success beyond efficacy	8 outcomes operationalise different dimensions: • Acceptability – stakeholder satisfaction with the innovation • Adoption – initial uptake or decision to use • Appropriateness – perceived fit for setting or problem • Feasibility – practicality within real‐world constraints • Fidelity – adherence to intended protocol • Implementation cost – total resources and expenses • Penetration – reach within eligible units or populations • Sustainability – maintenance over time in routine practice

CFIR: Consolidated Framework for Implementation Research.

ERIC: Expert Recommendations for Implementing Change.

IOF: Implementation Outcomes Framework.

Although systematic reviews have recently illuminated the numerous difficulties that impede the delivery of evidence-based nutritional care, they rarely investigate how specific implementation strategies influence the outcomes. In primary care, Zandonadi De Oliveira et al. (2021) mapped implementation steps and contextual determinants, while Launholt et al. (2025) extended that mapping to municipal home-care services for older adults; both stopping short of outcome evaluation. Chen et al. (2024) drew together community-based studies to portray perceived barriers and facilitators, and Raghunathan et al. (2024) did the same for maternity and neonatal units, again without linking strategies to effect sizes or patient endpoints. Even Li et al. (2025), whose combined qualitative and quantitative evidence in adult intensive care units focused on factors influencing guideline adherence rather than on downstream clinical or implementation outcomes. Collectively, these reviews identified the determinants influencing nutritional care but do not map the implementation strategies employed or evaluate their effectiveness.

This review aims to address this gap by synthesising and assessing current evidence on the implementation outcomes and clinical effectiveness of implementation strategies used to promote evidence-based nutritional care practices within healthcare settings.

## 2 Methods

A mixed-methods systematic review was conducted following the Joanna Briggs Institute (JBI) guidelines (Lizarondo et al., 2024). This type of review allows the inclusion of studies employing quantitative, qualitative and mixed methodologies, which is essential for a comprehensive evaluation of implementation outcomes based on the Implementation Outcomes Framework (Proctor et al., 2011). The Preferred Reporting Items for Systematic Reviews and Meta-Analyses (PRISMA) checklist was used to report the results of this study (Supplementary Material 1) (Page et al., 2021). While PRISMA encourages prospective protocol registration, the study was initially conceived as a scoping review, an approach not eligible for PROSPERO. Subsequent mapping and deliberation among the author team led to its refinement into a mixed-methods systematic review after data extraction had begun. Registering at that juncture would have been retrospective and potentially misleading; the review was not prospectively registered.

### 2.1 Study inclusion and exclusion criteria

Eligibility was structured using a combined PICO/PICo (Population; Intervention; Phenomenon of Interest; Outcomes; Context; Types of studies) framework appropriate to a mixed methods systematic review (Lizarondo et al., 2024). The full inclusion and exclusion criteria are presented in Table 2.

**Table 2:** Inclusion and exclusion criteria.

	Inclusion criteria	Exclusion criteria
**Population**	Studies including patients of any age with any health condition requiring nutritional care and/or their families	Studies conducted exclusively on healthcare students or where results cannot be distinguished from those of qualified professionals
	Studies including healthcare professionals involved in clinical practice, regardless of discipline or profession (doctors, nurses, or allied health professionals), as well as hospital administrators, senior/executive staff, or policymakers in health	
**Intervention**	Studies that defined implementation as an objective, goal, or outcome	Studies that did not provide an explicit description of implementation strategies
	Studies reporting at least one implementation strategy in the methods section	Studies that did not provide an explicit description of evidence-based nutritional care
**Phenomenon of interest**	Studies that investigated stakeholders’ perceptions, experiences, and processes related to at least one implementation outcome	
**Outcomes**	Studies that reported at least one implementation outcome measure within the results section	
**Context**	Studies focused on the implementation of evidence-based nutritional care in healthcare settings (hospital organizations and long-term care facilities)	Studies focused on public health, primary care, and family/general medicine
		Studies conducted outside the clinical context (in schools or social care services)
**Types of studies**	Peer-reviewed primary studies (quantitative, qualitative, or mixed methods)	Studies lacking a clear methodological section were excluded
	Companion papers published on the same intervention as included studies, focusing on other aspects of outcomes (determinants, real-world effectiveness, additional implementation outcomes)	Systematic reviews, theses, editorials, commentaries, and conference abstracts. References from these were reviewed if closely aligned with inclusion criteria in terms of population, phenomenon of interest, and context
	No restriction on the language of publication. Google Translate© and DeepL© were used to review titles and abstracts not in English or French. No linguistic expert was involved in the full-text review	

### 2.2 Search strategy

A comprehensive literature search was conducted across fifteen databases, covering January 2015 to January 2025. In accordance with the PRISMA-S extension guidelines (Rethlefsen et al., 2021), an initial exploratory search was performed in PubMed and CINAHL to identify relevant keywords, terms used in titles and abstracts, and indexing terms related to the topic. These were then organised into three core concepts: (1) evidence-based nutritional care practices; (2) implementation models and/or strategies; and (3) implementation outcomes. The initial search strategy was developed and piloted by JM in accordance with PRISMA-S extension for literature searches. Three reviewers (JA, SC, and ACA) reviewed and refined the full search strategy to ensure completeness and consistency.

The databases searched included: MEDLINE (PubMed), CINAHL (EBSCOhost), Embase (Elsevier), Cochrane Library (Wiley), Web of Science (Clarivate), Emcare (Ovid), JBI EBP Database (Ovid), ScienceDirect (Elsevier), HMIC (ProQuest), BNI (ProQuest), PsycINFO (EBSCOhost), BSC (EBSCOhost), EconLit (EBSCOhost), ERIC, and LiSSa. Boolean operators and controlled vocabulary were adapted to each database as appropriate (see Supplementary Material 2). No automated filters integrated into the databases were applied. In addition, backwards citation tracking was conducted by reviewing the reference lists of all included studies to identify any further relevant articles.

The search was restricted to publications from January 2015 to January 2025, with no language limitations to capture the state of the literature since the publication of two seminal implementation science contributions: Nilsen’s taxonomy of implementation theories, models, and frameworks (Nilsen, 2015); and the Expert Recommendations for Implementing Change project’s refined compilation of implementation strategies (Powell et al., 2015; Waltz et al., 2015). The search was not updated after January 2025.

### 2.3 Selection of studies

Search results were exported in RIS format, imported into Zotero® (Digital Scholarship, VA, USA) for duplicate removal, and subsequently transferred to SUMARI® (JBI, Adelaide, Australia) for screening. Two reviewers (JM and JA) independently screened titles, abstracts, and full texts based on predetermined inclusion and exclusion criteria. Any disagreements in selection decisions were resolved through discussion between the reviewers, with a third independent reviewer (ACA) consulted to achieve consensus if needed. One reviewer (JM) screened the references identified through citation tracking. Relevant articles were imported into Zotero® and SUMARI® and underwent the same selection process as database search results. Multiple publications reporting on the same study (companion papers) were identified and managed in accordance with JBI guidelines and the PRISMA statement. Only companion papers that met the inclusion criteria or provided additional data necessary for the analysis were retained. Selection outcomes and reasons for exclusion were reported in the PRISMA flow diagram (Page et al., 2021).

### 2.4 Data extraction

A standardised, integrated data extraction form was developed in Excel® (Microsoft, WA, USA) to systematically capture methodological details, participant characteristics, interventions, outcomes, and analyses from each included study. The two reviewers (JM and JA) independently pilot-tested the extraction process on a sample of five articles, and discrepancies were discussed to refine the extraction form. Subsequently, data extraction was performed by one reviewer (JM) and independently verified by a second reviewer (JA) to ensure reliability and consistency. Qualitative data, including themes, subthemes, barriers, and facilitators to implementation, were systematically extracted. Quantitative data related to implementation outcomes, quality-of-care outcomes, and patient-reported outcomes were also recorded when available. For companion papers, reports were grouped into clusters to facilitate integrated extraction. A primary report was selected as the principal data source, supplemented by complementary methodological details, secondary analyses, or additional results from companion papers. Consistency was verified across multiple reports, discrepancies were explicitly documented, and authors were contacted for clarification as necessary. This approach produced a comprehensive, unified dataset for each study, avoiding duplication and ensuring completeness.

### 2.5 Data synthesis and integration

A convergent segregated mixed-methods approach was applied (Lizarondo et al., 2024). Quantitative results were synthesised narratively because heterogeneity in study design, context and outcome metrics precluded meta-analysis. Qualitative evidence underwent a meta-aggregative narrative synthesis to derive categories that captured recurring contextual influences, implementation processes and perceived effects. Integration was achieved through configurative analysis: quantitative effect patterns were constantly compared with qualitative categories to build a coherent line of argument centred on implementation outcomes. Where direct configuration was not possible, findings are reported in parallel narrative form to maintain transparency.

All theories, models and frameworks cited in the primary studies were checked against the Wang et al.'s (2023) compendium; any unlisted theory, model, or framework was documented separately. To enable comparison across studies, implementation data were recoded onto three complementary frameworks: the Consolidated Framework for Implementation Research for determinants (Damschroder et al., 2022), the Expert Recommendations for Implementing Change strategies (Powell et al., 2015; Waltz et al., 2015), and the Implementation Outcomes Framework (Proctor et al., 2011).

Each framework provided a consistent set of constructs, and explicit criteria were applied for assigning study data to those constructs. Two reviewers (JM, JA) independently extracted and coded all data; a third reviewer (ACA) resolved any discrepancies.

### 2.6 Methodological quality assessment

The quality of included studies was independently assessed by two reviewers (JM and JA) using the Mixed-Methods Appraisal Tool (MMAT) (Hong et al., 2018). It was chosen because it allows quality evaluation across qualitative, quantitative, and mixed-methods studies. A third reviewer (ACA) was consulted when no consensus could be reached. Final scores were presented as percentages alongside extracted data in the tables, with a detailed overview of study scores provided in a separate table (Supplementary Material 3). No studies were excluded based on quality assessment; however, the results were considered in the discussion.

## 3 Results

### 3.1 Study selection

**Fig. 1:**
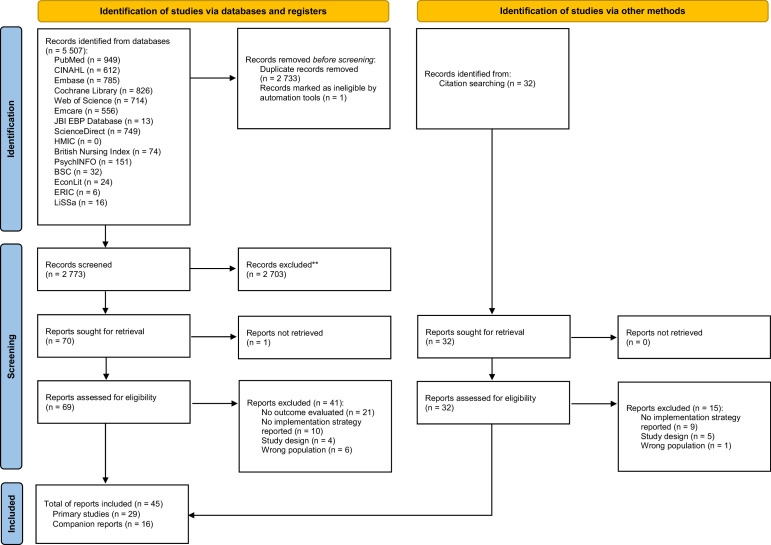
PRISMA flow diagram (Page et al., 2021).

As summarised in the PRISMA flow diagram (Fig. 1), systematic searches yielded 5539 records (5507 via databases; 32 from other sources). After duplicate removal, 2805 unique titles and abstracts were screened, and 101 full‐text articles were examined for eligibility. 29 primary studies met inclusion criteria. Additionally, 16 companion reports were collated to provide methodological detail or extended follow-up.

### 3.2 Characteristics of included studies

The 29 primary studies enrolled 1624 health-care professionals and 13,523 patients (details in Table 3). Two-thirds (*n* = 19) were published between 2018 and 2021, five each between 2015 and 2017, and between 2022 and early 2025. The work was predominantly Australian (16 studies), with smaller contributions from China (*n* = 4), Canada, Singapore and the United States (*n* = 2 each), and single studies from other countries.

**Table 3:** Summary of characteristics of included studies (*n* = 29).

	Number of studies (%)	Number of professionals	Number of patients
**Publication year**			
*2022–2025*	5 (17 %)	55	465
*2018–2021*	19 (66 %)	1160	12,555
*2015–2017*	5 (17 %)	409	503
**Country**			
*Australia*	16 (55 %)	328	2320
*China*	4 (14 %)	162	418
*Canada*	2 (7 %)	711	10,188
*Singapore*	2 (7 %)	26	108
*USA*	2 (7 %)	24	79
*Others*	3 (10 %)	373	410
**Methodology**			
*Quantitative, non-RCT*	20 (69 %)	1402	11,596
*Mixed*	8 (28 %)	193	1620
*Quantitative, RCT*	1 (3 %)	29	307
**Study design**			
*Monocentric*	23 (79 %)	624	2027
*Multicentric*	6 (21 %)	1000	11,496
*Before/After*	29 (100 %)	1624	13,523
**Specialty**			
*Surgery*	9 (31 %)	125	943
*Onco-hematology*	5 (17 %)	74	642
*Hospital*	5 (17 %)	818	11,250
*Pediatrics-Neonatal*	4 (14 %)	178	284
*Medicine*	3 (11 %)	160	147
*Intensive Care*	2 (7 %)	269	231
*Rehabilitation*	1 (3 %)	NR	26
**Targeted NCP step**			
*Intervention*	18 (62 %)	539	1741
*All steps*	8 (28 %)	885	11,340
*Assessment*	3 (10 %)	200	442
**Study duration**			
<6 months	5 (17 %)	128	344
6–12 months	7 (24 %)	148	681
12–24 months	10 (35 %)	1015	11,524
>24 months	7 (24 %)	333	974

RCT: Randomized Controlled Trial.

NCP: Nutrition Care Process.

NR: Not reported.

Clinical contexts spanned surgery (Byrnes et al., 2018; Colebatch and Lockwood, 2020; Den et al., 2021; Giuliani et al., 2015; Robertson et al., 2018; Seah et al., 2022; Takefala et al., 2024; Zhang et al., 2021; Zhang et al., 2021), general hospital settings (Bell et al., 2021; Gerrish et al., 2016; Keller et al., 2021a; 2019; Smith et al., 2018), and onco-haematology (Atkins et al., 2019; Findlay et al., 2020c; Loeliger et al., 2022; McCarter et al., 2018; Naseer et al., 2017). Other specialities included paediatric-neonatal (Costello, 2016; Cunha et al., 2024; Goodchild et al., 2018; Gu et al., 2020), medicine (Mackay et al., 2019; Sheng et al., 2020; Williams et al., 2019), intensive care units (Al Kalaldeh and Shahin, 2015; Barhorst et al., 2023), and rehabilitation (Mullins, 2021).

Regarding the nutrition care process, 62 % of studies focused exclusively on the intervention phase, 28 % addressed all care steps, and 10 % targeted assessment alone.

Methodologically, quantitative, non‐randomised before–and-after designs predominated (20 studies, 69 %), complemented by mixed‐methods approaches (8 studies) and a single randomised controlled trial. All studies employed a before-and-after design, with a majority being monocentric (23 studies, 79 %) rather than multicentric (6 studies). Study durations varied, with the most significant proportion lasting 12–24 months (10 studies, 35 %), while others spanned 6–12 months and over 24 months (7 studies each), and less than 6 months (5 studies).

Seven clusters of companion reports provided additional methodological detail or extended outcome data, ensuring internal validity by avoiding double‐counting: (1) Al Kalaldeh (2017) and Al Kalaldeh et al. (2015, 2014); (2) Byrnes et al. (2019); (3) Beck et al. (2021, 2020), Britton et al. (2019), and Murray et al.(2019); (4) Findlay et al. (2020b, 2021); (5) Laur et al. (2017, 2018a, 2018b, 2019); (6) Laur et al. (2021); and (7) Young et al. (2019). Treating these articles as linked companion reports prevents double-counting and maintains the internal validity of the evidence synthesis.

### 3.3 Quality appraisal

All 45 reports met the initial screening criteria and underwent full quality appraisal using the Mixed-Methods Appraisal Tool. Final quality scores (Supplementary Material 3) ranged from 80 % to 100 %. Overall, 11 studies (24 %) scored 80 %, 17 (38 %) scored 90 %, and 17 (38 %) achieved a perfect score of 100 %. The multifaceted and tailored nature of the interventions, combined with the action-research approach in many studies, frequently made it difficult to ensure outcome assessor blinding or to account for confounders in design and analysis fully.

### 3.4 Implementation theories, models and/or frameworks used

Across the included studies, 86 % explicitly referenced an implementation theory, model or framework: 52 % employed one, while 35 % combined two or more approaches (Table 4; see Supplementary Material 3). The application of these models reflected a nuanced approach to guiding implementation, often aligning with recommendations to tailor and operationalise frameworks throughout the implementation process.

**Table 4:** Overall summary of key implementation elements described in the included reports.

Process models were the most frequently utilised (69 %), serving as structured guides for translating evidence into practice. The Joanna Briggs Institute Model of Evidence-Based Healthcare was applied in 4 % of studies supporting systematic processes including initial situational analysis, targeted strategy design, and iterative audit-feedback cycles to sustain change. The Knowledge to Action framework, present in 28 % of studies, provided a conceptual foundation for moving research into practice by informing barrier assessment, contextual adaptation, intervention design, and outcome evaluation. These models were often operationalised through established toolkits to facilitate practical application across implementation stages.

Determinant frameworks were employed in 41 % of studies to identify contextual, organisational, and individual factors influencing outcomes. The Theoretical Domains Framework was used in 21 % of cases to diagnose barriers and facilitators, frequently informing the subsequent tailoring of interventions via complementary models such as the Behaviour Change Wheel. The Promoting Action on Research Implementation in Health Services (PARIHS)/integrated-PARIHS framework (17 %) and the Consolidated Framework for Implementation Research (14 %) provided comprehensive approaches to assess context, recipient characteristics, and facilitation strategies, and were sometimes combined to enhance contextual adaptation and strategy selection. 14 % of studies featured theories focused on behaviour change, with the Capability, Opportunity, Motivation and Behaviour (COM-B) model (10 %) and Normalisation Process Theory (7 %) primarily used to analyse determinants of individual behaviour, inform the design of targeted interventions, and understand the embedding of new practices within routine care.

Evaluation frameworks were cited in 10 % of studies, notably the Implementation Outcomes Framework (7 %) for standardised measurement of key outcomes, and the Theoretical Framework of Acceptability (3 %) to assess stakeholder acceptance. In contrast, no study incorporated traditional or classic theories.

Collectively, the selected models were integrated at multiple phases of the implementation process, either individually or synergistically, to optimise contextual fit, specify mechanisms of change, and strengthen both the design and evaluation of implementation strategies.

### 3.5 Identified implementation barriers and facilitators

A comprehensive analysis of our implementation study revealed 158 distinct barriers and 79 facilitators distributed across all four Consolidated Framework for Implementation Research domains (Table 3; see Supplementary Material 3 for full details).

At the level of intervention characteristics, the inherent complexity of the Nutrition Care Pathway (*n* = 5) emerged as a recurrent obstacle. Ward teams found themselves repeatedly tailoring protocols before the pathway could be enacted within busy, variable workflows. Moving into the inner setting, three intertwined constraints stood out. First, resource limitations (*n* = 18) were stark: high nursing turnover led to continual educational refresher sessions, while the lack of dietetic support outside standard hours forced clinicians to postpone critical feeding decisions until the next business day. Second, communication inconsistencies (*n* = 13) bred confusion. Parallel paper and electronic systems sometimes recorded conflicting diet orders, and crucial nutrition goals were often omitted from interdisciplinary handovers, leaving care teams with divergent plans. Third, inadequate infrastructure (*n* = 13) further hampered progress; bedside weighing scales were unavailable on several units, and no clear, multidisciplinary workflow existed to expedite enteral feeding initiation, resulting in protracted approval loops.

Additional inner-setting barriers: limited access to up-to-date protocols (*n* = 7), cultural resistance to early feeding (*n* = 6), misalignment with existing workflows (*n* = 6), combined with a low tension for change (*n* = 5), reflecting widespread clinician scepticism about the urgency of early nutrition interventions.

Turning to the individual characteristics domain, capability deficits were observed (*n* = 18) in which many clinicians lacked confidence or sufficient knowledge to conduct malnutrition screening reliably. Alongside this, low motivation (*n* = 6) surfaced as a barrier when competing clinical priorities led staff to deprioritise nutrition tasks. Finally, within the process domain, insufficient planning (*n* = 7) hindered the establishment of robust feedback loops and progress monitoring, undermining the pathway’s sustainability over time.

Yet, these barriers coexisted with notable facilitators. The evidence base underpinning early nutrition (*n* = 7) instilled confidence in screening and feeding protocols. A nascent pro-nutrition culture (*n* = 5) saw wards celebrate “feed-within-six-hours” achievements, reinforcing shared ownership. Standardised communication pathways (*n* = 8), such as a unified electronic handover template, fostered clarity and consistency. Mid-level leaders (*n* = 6), particularly nurse champions mediating between dietitians and physicians, galvanised staff buy-in. Structured planning (*n* = 8) via iterative Plan–Do–Study–Act cycles, coupled with routine reflection and evaluation (*n* = 8) through monthly performance dashboards, maintained momentum. Lastly, active engagement from nursing and physician leaders (*n* = 7) and tailored, unit-specific strategies (*n* = 6) ensured the pathway remained feasible, acceptable, and sustainable.

### 3.6 Implementation strategies used

Across the included studies, implementation strategies were predominantly multifaceted and contextually tailored. Five studies employed multifaceted approaches, while 24 studies used tailored, multifaceted strategies; notably, no study relied on a single strategy (Table 3; see Supplementary Material 3 for details).

#### 3.6.1 Train and educate stakeholders

Education-based strategies were among the most frequently reported. A total of 97 % of studies implemented “conduct educational meetings,” typically delivered by local champions, principal investigators, or interdisciplinary project teams. These meetings, targeting nurses, allied health professionals, and multidisciplinary care units, aimed to address commonly cited educational deficits, particularly limited knowledge of new clinical guidelines or best practices. Delivery formats varied from intensive, one-off workshops to regularly scheduled in-service sessions over several months. This temporal flexibility allowed implementers to accommodate both initial training needs and ongoing reinforcement.

In 55 % of studies, these meetings were complemented using “develop educational materials,” including handouts, posters, and manuals, which served as standardised tools to reinforce key messages and sustain knowledge dissemination beyond face-to-face interactions. Additionally, 21 % of studies incorporated “conduct ongoing training” as a sustained educational approach, including booster sessions, periodic refreshers, or individualised coaching. These strategies were often employed to support skill retention over time, mitigate the impact of staff turnover, and promote adherence to newly adopted practices.

#### 3.6.2 Use evaluative and iterative strategies

Evaluative and iterative strategies were also widely applied. “Audit and provide feedback” appeared in 93 % of studies, with audits typically performed by research staff or trained clinical champions through systematic reviews of patient charts, electronic medical records, or direct observation. These feedback cycles (ranging in frequency from weekly to monthly) were used to monitor fidelity, identify performance gaps, and support continuous quality improvement. Their iterative use across multiple implementation phases (baseline, mid-point, post-implementation) underscored a commitment to real-time adaptation and learning.

Additionally, 83 % of studies included both “purposely reexamining the implementation” and “tailoring strategies,” frequently deploying them in tandem. These strategies enabled clinical teams to reflect on process outcomes, adapt interventions to emerging findings, and align implementation approaches with local barriers and facilitators. The deliberate use of tailoring was often informed by preliminary assessments, as 76 % of studies reported “assess for readiness and identify barriers and facilitators.” These assessments evaluated organisational culture, leadership dynamics, and resource availability, enabling more context-specific and effective strategy design.

#### 3.6.3 Develop stakeholder interrelationships

Another critical feature was the development of collaborative structures and relational dynamics between stakeholders. Eighteen studies (62 %) reported the use of “use advisory boards and workgroups,” involving multidisciplinary teams (nurses, dietitians, physicians, and managers) to inform implementation planning and guide local decision-making. These groups served as key forums for co-designing protocols, contextualising guidelines, and negotiating shared ownership of implementation goals. The frequency of engagement varied by project complexity, with meetings held weekly, bi-monthly, or monthly.

In parallel, six studies (21 %) employed “identify and prepare champions,” often selecting individuals with clinical credibility or peer influence. These champions typically received specialised training or mentorship and were tasked with promoting the intervention, addressing resistance, and maintaining engagement over time. When used together, advisory structures and champions fostered alignment across stakeholder perspectives, strengthened organisational commitment, and increased the likelihood of sustained behaviour change.

#### 3.6.4 Provide interactive assistance

Interactive assistance strategies further enhanced implementation by offering real-time, practice-oriented support. “Facilitation” was reported in 24 % of studies, operationalised through the involvement of internal or external facilitators who provided sustained assistance via on-site visits, teleconferences, or email communication. Facilitators were key in troubleshooting challenges, reinforcing training content, and maintaining implementation momentum. Their support was often embedded in structured follow-ups (monthly coaching calls or community-of-practice sessions), emphasising the importance of ongoing engagement beyond initial rollout.

Additionally, 21 % of studies used “remind clinicians” as a reinforcement tool. This involved delivering prompts (such as posters, checklists, or electronic alerts) designed to increase the salience of desired behaviours within daily routines.

#### 3.6.5 Change infrastructure

Although less commonly reported, structural changes played a critical role in embedding new practices into existing systems. Eight studies (28 %) reported “change record systems,” involving integrating screening tools, alert mechanisms, or templates into electronic medical records. These system-level adaptations were designed to streamline documentation, automate referrals, and facilitate adherence to nutritional care processes. Such changes required coordination with information technology and administrative departments and often included pilot testing before broader implementation.

Three studies (10 %) also utilised “change physical structure and equipment,” introducing modifications to the clinical environment (such as standardised supply kits or reorganised storage systems) to enhance workflow efficiency and support protocol adherence.

#### 3.6.6 Financial strategies

Finally, financial strategies were implemented to a limited extent but were critical enablers in resource-constrained settings. Three studies (10 %) reported “access new funding,” typically securing small grants or dedicated staff support to offset training, data collection, or implementation-related labour costs. In one study (3 %), “alter incentive/allowance structures” was employed through non-financial rewards distributed at key project milestones. These approaches addressed financial and logistical barriers and were often justified by investigators as necessary to ensure stakeholder engagement and maintain momentum when internal resources were insufficient.

### 3.7 Effects of implementation strategies

All studies evaluated at least one implementation outcome, seventeen assessed service outcomes and seven reported patient outcomes (Table 3, Table 4; details in Supplementary Material 3). Fourteen of these investigations also generated qualitative data enriching interpretation of the quantitative effects (Table 5).

**Table 5:** Overall summary of main outcomes described in the included studies (*n* = 29).

Outcomes	Studies; *n* = (%)	Key findings	Overall impact	Limitations	Significance
***Implementation outcomes***					
*Acceptability*	7 (24 %)	High acceptability (70 %+ positive feedback), strong staff support	Strong	Metrics mainly descriptive	Some studies report p-values (*p* < .05)
*Adoption*	1 (3 %)	79 % adoption among targeted clinicians	Moderate to Strong	Limited to one study	Not reported
*Appropriateness*	1 (3 %)	Improved staff confidence: 89 % of patients deemed appropriate for intervention	Strong	Limited to one study	Not reported
*Feasibility*	2 (7 %)	Improved from 46 % to 73 %; logistical barriers noted	Moderate	Mixed feasibility outcomes	One study report p-values (*p* < .001)
*Fidelity*	29 (100 %)	High adherence rates (>80–100 %), significant improvements	Strong	Rarely reported confidence intervals	Many studies report p-values (*p* < .05 or *p* < .001)
*Implementation cost*	7 (24 %)	Cost-benefit favorable, resource use varied	Moderate to Strong	Not consistently reported	Some studies report p-values (*p* < .05)
*Penetration*	3 (10 %)	Interventions integrated across multiple sites, roles	Moderate	Limited studies reporting	Not reported
*Sustainability*	3 (10 %)	Long-term compliance (minor declines but sustained improvements)	Moderate to Strong	Metrics mainly descriptive	Some studies report a p-values (*p* < .05), but formal p-values rare
***Service outcomes***					
*Timeliness of nutrition care*	6 (21 %)	Faster initiation of enteral nutrition, reduced fasting times	Strong	Consistent findings across studies	Many studies report p-values (*p* < .05 or *p* < .01)
*Nutrition coverage & adequacy*	10 (34 %)	Nutritional adequacy improved (50 % to 80–100 %), better screening rates	Strong	Rarely reported confidence intervals	Many studies report p-values (*p* < .05 or *p* < .001)
*Treatment completion & interruption*	3 (10 %)	Fewer interruptions, higher completion rates	Moderate to Strong	Limited studies reporting	Some studies report p-values (*p* < .05 or *p* < .001)
*Clinical complications*	6 (21 %)	Reduced vomiting (from 42 % to 21 %), lower infection rates, etc.	Moderate to Strong	Rarely reported confidence intervals	Many studies report p-values (*p* < .05)
***Patient outcomes***					
*Nutritional status & body weight loss*	4 (14 %)	PG-SGA scores improved; weight loss decreased	Moderate to Strong	Limited studies reporting	Many studies report p-values (*p* < .05 or *p* < .01)
*Quality of life & mental health*	1 (3 %)	Improved depression scores and quality of life gains	Moderate	Limited to one study	Study report p-values (*p* < .05, *p* < .01)
*Patient satisfaction, empowerment & experience*	3 (10 %)	Higher satisfaction, improved self-monitoring, better awareness of malnutrition risks	Moderate	Limited studies reporting	One study report p-values (*p* < .01), others descriptive

PG-SGA: Patient-Generated Subjective Global Assessment.

#### 3.7.1 Effects of implementation strategies on implementation outcomes

Acceptability was reported in eight studies (28 %), which consistently demonstrated high levels of approval among healthcare professionals; more than 70 % of them expressed positive perceptions of the nutrition interventions. Qualitative analyses further highlighted strong healthcare staff endorsement of the new models of care and underscored the centrality of multidisciplinary collaboration and visible clinical leadership in fostering staff engagement. Adoption, evaluated in a mixed-methods study, reached 79 % among targeted clinicians; interview data attributed this uptake to the influence of clinical champions and the presence of audit-feedback mechanisms that reinforced intrinsic motivation. Appropriateness, assessed in one study, demonstrated an 89 % alignment between patient needs and the intervention protocol, with qualitative feedback emphasising the intervention’s congruence with existing clinical workflows.

Feasibility was addressed in two studies: while one identified logistical barriers such as limited access to appropriate food options outside standard hours, another documented a significant improvement in staff confidence, increasing from 46 % to 73 % (*p* < .001) following the introduction of a mobile nutritional assessment tool. Fidelity was reported in all 29 studies (100 %), emerging as a consistently robust outcome; adherence rates commonly exceeded 80 %, with several instances reaching 100 %. These high levels of fidelity were supported by qualitative and observational data, which linked adherence to ongoing real-time monitoring and the modelling of best practices by senior clinicians.

Implementation cost was evaluated in seven studies (24 %), with most reporting favourable cost–benefit profiles at the health-service level. Reported economic impacts ranged from modest increases in staff expenditure to notable health-service cost savings, exemplified by a net saving of AUD $14.65 for every AUD $1 invested and an avoided annualised expenditure of AUD $121,100. Penetration was assessed in three studies (10 %), revealing successful expansion of nutritional care strategies across multiple clinical units or professional roles, as demonstrated by 45 % of participants receiving specialised dietitian or physiotherapist input. Sustainability, explored in four studies (14 %), indicated that improvements were generally maintained over extended periods; however, some studies documented minor reductions in compliance rates over time (from 84 % to 63 %).

#### 3.7.2 Effects of implementation strategies on service outcomes

Timeliness of nutrition care was evaluated in six studies (21 %), showing marked improvements. For example, the median time to initiation of enteral nutrition was reduced from 3.15 days to approximately one day (*p* < .05), and rates of early nutrition support increased from 53 % to 79 % (*p* < .01). Qualitative accounts linked these gains to the streamlining of care pathways and the appointment of dedicated coordinators.

Nutritional coverage and adequacy were addressed in ten studies (34 %), where targeted intake increased from approximately 50 % to between 80 % and 100 %. Advanced nutrition care processes expanded from 30 % to over 60 %, and comprehensive nutrition assessments rose from 24 % to 83 % (*p* < .001). Complementary qualitative evidence identified improved interprofessional communication and digital dashboards to monitor patient progress as key facilitators.

Three studies (10 %) investigated treatment completion or interruption, reporting substantial reductions in feeding interruption durations (median decrease of 12.7 h, *p* < .001), a decrease in the proportion of patients experiencing treatment interruptions (from 14 % to 8 %, *p* < .05), and higher rates of completion for interventions such as radiotherapy or systemic therapy (from 67 to 89 % to 100 %, *p* < .05 or *p* < .005). Staff perspectives attributed these improvements to clearer diet-code definitions and enhanced consensus decision-making.

Six studies (21 %) evaluated service-level safety outcomes, assessed via patient complication rates, documenting reductions in vomiting among patients (from 42 % to 21 %, *p* < .05), decreases in gastrointestinal complications (from 38 to 30 events, *p* < .05), and declines in patient nutrition-related incidents (from 22 % to 9 %). These findings were corroborated by nurses and physicians, who perceived that proactive, protocol-driven care and shared accountability enhanced overall patient safety. Nevertheless, confidence intervals were infrequently reported, and descriptive statistics predominated in the quantitative analyses.

#### 3.7.3 Effects of implementation strategies on patient outcomes

Four studies (14 %) examined nutritional status or weight as patient outcomes, including both quantitative measurements and patient or caregiver interviews. Early specialist involvement and the provision of clearer information were reported to alleviate anxiety and mitigate weight loss. Among these studies, one reported a median weight loss of –3.3 kg, significantly less than in controls (–5.9 kg; *p* < .05), and Patient-Generated Subjective Global Assessment scores showed improvement or deterioration depending on context, with one trial demonstrating better nutritional scores (β = –1.53; 95 % CI −2.93 to −0.13) and one post-implementation cohort showing a slight worsening, potentially due to a higher proportion of high-risk surgical patients. Another study found better Subjective Global Assessment scores in the intervention group (14.71 vs. 16.24, *p* < .05), with 84 % of intervention patients classified as well-nourished compared to 87 % in controls (*p* < .01). Overall, these findings indicate that nutritional care was effective in improving nutritional status and limiting weight loss, with context-dependent variation.

Quality of life and mental health outcomes were assessed in a single study, with patient testimonies highlighting the benefits of ongoing supportive counselling. The intervention group reported significantly lower depression scores (5.79 vs. 6.68, *p* < .05) and higher health-related quality of life (0.45, *p* < .01).

Three studies (10 %) evaluated patient satisfaction, empowerment, and experience by integrating survey data and qualitative feedback. These studies reported improvements in patient satisfaction (ranging from 2 % to 18 %), increased self-monitoring of fluid intake, and greater awareness of malnutrition risk (from 24 % to 28 %). Additional significant increases were noted in the proportion of patients receiving nutrition information (from 15 % to 24 %) and actively participating in ongoing discussions about their nutrition plans (from 11 % to 26 %; all *p* < .01).

## 4 Discussion

This systematic review synthesised evidence from 29 primary studies evaluating implementation strategies designed to promote evidence-based nutritional care across diverse healthcare settings. It goes beyond prior reviews that predominantly catalogue barriers and facilitators by explicitly linking these determinants to implementation strategies employed and their measurable effects on implementation and clinical outcomes.

Our findings indicate that multifaceted, contextually tailored implementation strategies, combining audit and feedback, educational outreach, leadership engagement, and system-level modifications, are associated with consistently high fidelity, strong acceptability, and improvements in feasibility. These strategies contributed to enhanced service outcomes, including more timely initiation of nutrition therapy, increased nutritional adequacy, reduced complications, and patient benefits such as reduced weight loss and improved nutritional status.

**Fig. 2:**
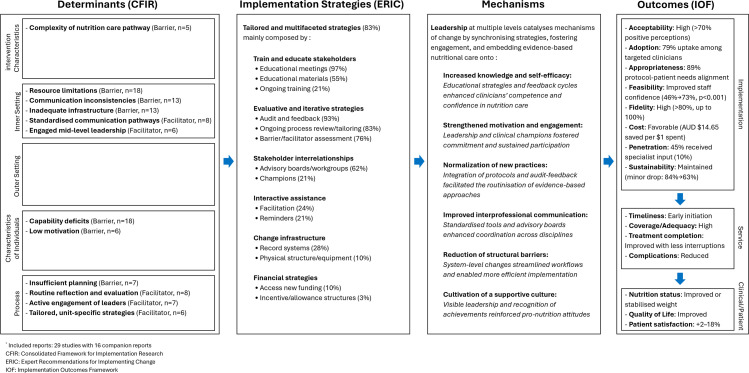
Fig. 2. Overview of the evidence-based nutritional care implementation process reported in included reports, synthesised using the Implementation Research Logic Model (Smith et al., 2020).

Fig. 2 summarises these findings within the Implementation Research Logic Model (Smith et al., 2020), mapping key contextual determinants (resource constraints, communication challenges), implementation strategies (education, audit-feedback, stakeholder engagement), and their underlying mechanisms to observable implementation and clinical outcomes.

### 4.1 Theoretical frameworks: use and gaps

The use of theoretical models across the included studies was marked by considerable heterogeneity, reflecting broader trends previously identified in implementation research (Strifler et al., 2018; Wang et al., 2023). Most primary studies provide little to no rationale for their chosen models, reflecting a tendency for researchers to default to familiar frameworks rather than employ a deliberate selection process that aligns with their specific objectives, context, and evidence requirements (Barnden et al., 2023; Birken et al., 2017).

The included studies favoured process models for structuring the stepwise translation of evidence into practice. Determinant frameworks were frequently used to diagnose barriers and facilitators, often in tandem with process models. When fully integrated, this combination can create a coherent pathway from contextual assessment to strategy design (Moullin et al., 2020). Evaluation frameworks were infrequently invoked, perpetuating a disconnect between implementation processes and the rigorous assessment of outcomes (Proctor et al., 2023).

To advance theoretical coherence, emerging decision aids such as the Theory Comparison and Selection Tool (Birken et al., 2018b) and the more recent SELECT-IT meta-framework (Fontaine et al., 2025) offer structured criteria to align theoretical models’ attributes with project aims and contextual constraints.

### 4.2 Contextual determinants

The barriers identified in this review align closely with those reported on the broader literature, highlighting consistent implementation challenges across various healthcare settings. Similar to our findings, resource constraints, including high staff turnover and insufficient workforce support, have been recognised as significant obstacles to sustaining nutrition interventions, particularly in intensive care and aged-care environments (Launholt et al., 2025; Li et al., 2024). Communication inconsistencies and inadequate infrastructure disrupting coordination have also been widely reported, underscoring their pervasive impact on clinical practice (Launholt et al., 2025; Li et al., 2024; Raghunathan et al., 2024).

Our analysis further supports previous findings at the individual level, identifying capability deficits, low motivation, and clinician scepticism as critical barriers. Similarly, Chen et al. (2024) emphasis should be placed on the importance of individual skill-building and motivation, alongside interprofessional collaboration, to overcome these barriers, particularly among older adults experiencing frailty. Additionally, issues surrounding cultural compatibility and limited access to nutrition protocols observed in our study reflect challenges previously reported by Launholt et al. (2025) and Li et al. (2024), who emphasise the necessity of aligning evidence-based recommendations with local norms and clinical contexts.

Facilitators identified in our synthesis also align well with existing literature, reinforcing the role of robust evidence, structured communication strategies, mid-level leadership, and tailored implementation plans in promoting sustainable nutritional care. Consistent with previous findings (Chen et al., 2024; Raghunathan et al., 2024), structured planning, continuous evaluation, and active engagement of healthcare leaders have emerged as critical components for enhancing staff buy-in and achieving sustained improvements in practice.

### 4.3 Tailored and multifaceted strategies

The findings from the included studies underscore the complexity of implementing nutrition research in real-world settings (Cederholm et al., 2017; Findlay et al., 2020a), highlighting the necessity for tailored implementation strategies. These strategies must be carefully adapted to local contexts by addressing specific barriers and facilitators, as generic, one-size-fits-all approaches have shown limited effectiveness. Consistent with prior research (Boaz et al., 2024; Waltz et al., 2019), evidence suggests that the “fit” between an implementation strategy and the contextual challenges it targets is a stronger predictor of both practitioner behaviour and patient outcomes than simply the type or number of strategies employed (Chays‐Amania et al., 2025). Tailoring strategies to the nuances of local determinants enables more precise and effective promotion of evidence-based nutritional care.

Complementing the emphasis on tailoring, the studies collectively point to the value of multifaceted strategies, wherein multiple interventions are combined to address complex implementation challenges. While deconstructing the unique contribution of each component within a multifaceted intervention remains difficult (Ashcraft et al., 2024), the aggregated evidence nonetheless shows that programmes combining multiple, context-relevant strategies tend to yield more favourable outcomes than those implemented in isolation (Fontaine et al., 2024; Spoon et al., 2020).

### 4.4 Leadership-driven implementation process

Across the included studies, leadership consistently emerges as the essential conduit for implementing evidence-based nutritional care into practices. Interpreted through the Implementation Leadership Model (Aarons et al., 2014), their findings show that distinct leadership roles catalyse identifiable mechanisms of change that, in turn, sustain a coherent bundle of strategies in the implementation process.

Champions act as front-line change agents who disseminate guidelines, model desired behaviours and iteratively tailor interventions to local contingencies; their peer credibility and change-management skills explain much of their influence (Laur et al., 2021). Advanced Practice Nurses, by virtue of their extended clinical expertise and cross-disciplinary authority, can represent an especially promising champion profile (Schwingrouber et al., 2024). Middle managers, meanwhile, bridge strategic intent and operational reality by translating organisational goals into executable plans, brokering information vertically and aligning resources horizontally; these activities shape an implementation climate that normalises evidence use (Birken et al., 2018a). Although absent from the reviewed studies, evidence-based practice mentors focus on nurturing a sustained culture of inquiry and methodological competence across clinical teams (Agnel et al., 2025).

The Implementation Leadership Model clarifies how these roles exercise their influence. Champions exemplify proactive leadership when they anticipate barriers and sequence rollouts of nutritional care, whereas middle managers display supportive leadership by securing staff or budgetary resources; both roles require knowledgeable command of the evidence base and sustained perseverance to maintain momentum amid competing priorities (Birken et al., 2018a; Laur et al., 2021).

Five strategic components recur across studies and depend on the foregoing mechanisms for their effectiveness. Sequenced educational interventions delivered chiefly by champions incrementally raise staff competence without disrupting workloads (Jolliffe et al., 2025). Audit-and-feedback cycles provide timely performance data that trigger adaptive refinements and consolidate accountability (Snider et al., 2023). Through high-intensity, relationship-focused interactions, facilitation offers real-time problem-solving and tailoring implementation strategies to specific contextual demands (Lizarondo et al., 2023). Forming advisory boards and workgroups engages interdisciplinary stakeholders in co-designing contextually appropriate solutions, thereby legitimising diverse forms of knowledge and reinforcing stakeholder commitment (Santos et al., 2022). Infrastructural adjustments, championed by leaders, dismantle structural impediments and embed novel organisational routines (Williams et al., 2020). Leadership thus does more than authorise these strategies; it synchronises their sequencing, ensures mutual reinforcement and maintains momentum, securing long-term sustainability.

#### 4.4.1 Strengths and limitations

This review has several methodological strengths. It followed JBI guidance and PRISMA, enhancing transparency and reproducibility. A sensitive search of numerous bibliographic databases, with forward- and backward-citation tracking, reduced publication, language, and indexing bias. Study selection, data extraction, and quality appraisal were performed in duplicate using the Mixed-Methods Appraisal Tool; companion papers were clustered a priori to maximise completeness and minimise reviewer bias. Quantitative and qualitative evidence were integrated within a coherent, theory-driven framework, enabling nuanced analysis of how implementation strategies interact with contextual determinants across heterogeneous clinical settings.

Several limitations temper confidence in these findings. Marked clinical and methodological heterogeneity in strategies, designs, settings, and outcome metrics, intrinsic to implementation science, limited quantitative pooling. Consequently, findings were integrated through structured narrative synthesis in line with best-practice guidance when meta-analysis is inappropriate. Although guided by explicit criteria and checked by independent reviewers, necessary reclassification and reformulation introduced subjectivity that may affect comparability. Reporting quality in many primary studies was uneven, with sparse detail on dose, fidelity, and operationalisation, hampering attribution and limiting reproducibility. Co-interventions and parallel quality-improvement initiatives were frequent yet incompletely described, complicating causal inference and constraining generalisability. Follow-up was often brief or inconsistently defined, restricting assessment of longer-term sustainability. Finally, despite frequent reference to theoretical frameworks, inconsistencies in selection and reporting reduced theoretical transparency and may have influenced the interpretation of underlying mechanisms.

#### 4.4.2 Recommendations

##### 4.4.2.1 Recommendations for practice

According to JBI (Lizarondo et al., 2024), recommendations are graded as Grade A (“strong”) when high‐quality evidence clearly demonstrates that desirable effects outweigh undesirable effects with minimal resource impact and patient values taken into account, and Grade B (“weak”) when evidence may be of lower quality or less conclusive regarding benefits, resource use, or stakeholder preferences. The findings of this systematic review suggest that clinicians and healthcare organisations seeking to enhance evidence-based nutritional care practices should use multifaceted and tailored implementation strategies (Grade A). In particular:

○ Educational initiatives should incorporate structured training programs (periodic interdisciplinary workshops and targeted booster sessions) to maintain clinician competence and address knowledge gaps identified as key barriers (Grade B).
○ Audit and feedback mechanisms should be operationalised through regular, structured reviews of clinical practice data (monthly or quarterly performance dashboards) to inform iterative practice improvements and sustain high levels of fidelity (Grade A).
○ Leadership engagement strategies should explicitly identify and train clinical champions and middle managers in specific skills related to change management and interprofessional collaboration, enabling them to lead implementation processes effectively (Grade B).
○ System-level modifications should include integrating standardised nutrition screening and monitoring tools into existing electronic medical records systems, and ensuring essential equipment and infrastructure (bedside scales, streamlined documentation workflows) are reliably available to facilitate timely and consistent practice (Grade B).

##### 4.4.2.2 Recommendations for research

The results of this review highlight several critical areas for further research to improve methodological consistency, rigour, and actionable knowledge within nutritional implementation science:

○ Adopt standardised methodologies by employing established, validated frameworks consistently across studies. Researchers should justify their choice of framework and comprehensively report how it informed each stage of the implementation process, from barrier assessment and strategy tailoring to outcome evaluation.
○ To elucidate the mechanisms through which tailored strategies operate, robust quantitative analytical methods should be incorporated alongside detailed qualitative assessments of local contextual factors.
○ Conduct studies with extended follow-up periods (minimum 12–24 months post-implementation), assessing sustainability, cost-effectiveness, and penetration.

## 5 Conclusion

This review demonstrates that multifaceted, contextually tailored implementation strategies (especially those combining education, audit and feedback, leadership engagement, and system-level changes) are associated with improved implementation, service and patient outcomes across healthcare settings. Our synthesis highlights the importance of aligning strategies with local determinants and engaging clinical leadership to drive sustainable practice change. The structured use of established implementation science frameworks enhanced analytic clarity, allowing for a nuanced understanding of the complex interplay between context, intervention components, and outcomes.

Nevertheless, methodological heterogeneity, variable reporting quality, and inconsistent application of theoretical frameworks remain significant limitations, complicating attribution of effects to specific strategies. Future research should prioritise the transparent, consistent application of validated frameworks, robust mixed-methods evaluation, and extended follow-up to assess sustainability and impact.

## Funding

This research did not receive any specific grant from funding agencies in the public, commercial, or not-for-profit sectors.

## Data availability

All data extracted during this mixed-methods systematic review are available in the published article and its Supplementary Files. Additional extraction sheets or analytic codes can be obtained from the corresponding author upon reasonable request.

## CRediT authorship contribution statement

**Jerome Molle:** Writing – original draft, Methodology, Investigation, Formal analysis, Data curation, Conceptualization. **Joris Agnel:** Writing – review & editing, Visualization, Validation, Methodology, Investigation. **Sebastien Colson:** Writing – review & editing, Project administration, Methodology. **Audrey Chays-Amania:** Writing – review & editing, Supervision, Methodology.

## Declaration of competing interest

The authors declare that they have no known competing financial interests or personal relationships that could have appeared to influence the work reported in this paper.

## Appendix Supplementary materials

Image, application 1

Image, application 2

Image, application 3
